# Doxycycline Inducible Kruppel-Like Factor 4 Lentiviral Vector Mediates Mesenchymal to Epithelial Transition in Ovarian Cancer Cells

**DOI:** 10.1371/journal.pone.0105331

**Published:** 2014-08-19

**Authors:** Zixuan Chen, Yinan Wang, Wen Liu, Guannan Zhao, Suechin Lee, Andrea Balogh, Yanan Zou, Yuqi Guo, Zhan Zhang, Weiwang Gu, Chengyao Li, Gabor Tigyi, Junming Yue

**Affiliations:** 1 Department of Pathology and Laboratory Medicine, University of Tennessee Health Science Center, Memphis, Tennessee, United States of America; 2 Center for Cancer Research, University of Tennessee Health Science Center, Memphis, Tennessee, United States of America; 3 Department of Physiology, University of Tennessee Health Science Center, Memphis, Tennessee, United States of America; 4 Southern Medical University, Guangzhou, P. R. China; 5 The Third Affiliated Hospital, Zhengzhou University, Zhengzhou, P. R. China; 6 The Second Affiliated Hospital of Harbin Medical University, Harbin, P. R. China; University of Kentucky, United States of America

## Abstract

Ovarian cancer presents therapeutic challenges due to its typically late detection, aggressive metastasis, and therapeutic resistance. The transcription factor Krüppel-like factor 4 (KLF4) has been implicated in human cancers as a tumor suppressor or oncogene, although its role depends greatly on the cellular context. The role of KLF4 in ovarian cancer has not been elucidated in mechanistic detail. In this study, we investigated the role of KLF4 in ovarian cancer cells by transducing the ovarian cancer cell lines SKOV3 and OVCAR3 with a doxycycline-inducible KLF4 lentiviral vector. Overexpression of KLF4 reduced cell proliferation, migration, and invasion. The epithelial cell marker gene E-cadherin was significantly upregulated, whereas the mesenchymal cell marker genes vimentin, twist1and snail2 (slug) were downregulated in both KLF4-expressing SKOV3 and OVCAR3 cells. KLF4 inhibited the transforming growth factor β (TGFβ)-induced epithelial to mesenchymal transition (EMT) in ovarian cancer cells. Taken together, our data demonstrate that KLF4 functions as a tumor suppressor gene in ovarian cancer cells by inhibiting TGFβ-induced EMT.

## Introduction

Ovarian cancer has a high mortality rate, reportedly causes 15,000 deaths annually in the US [1]. Although significant improvements have been made in the detection of ovarian cancers in the past decade, more than 20,000 new cases are diagnosed every year [1], [2]. The therapeutic options for ovarian cancer are limited because of its resistance to chemo- and radiation therapy leading to frequent recurrences [3], [4].

KLF4 has been shown to regulate cell proliferation and differentiation, its role has been extensively investigated in several human cancers by using gain- and loss-of-function approaches. In colon and prostate cancer, KLF4 acts as an oncogene [5], [6]. In contrast, it plays a tumor suppressor role in neuroblastoma, lung cancer, gastric cancer, lymphoma, cervical cancer, pancreatic ductal cancer, and hepatocellular carcinoma [7], [8], [9], [10], [11], [12], [13]. In breast cancer, KLF4 can function both as an oncogene [14], [15] and a tumor suppressor [16], [17], [18]. The role of KLF4 in ovarian cancer has not been adequately and mechanistically addressed. A previous study indicates that the expression level of KLF4 was significantly reduced in ovarian cancer compared to normal ovarian epithelium, suggesting that KLF4 might potentially act as a tumor suppressor in ovarian cancer [19].

KLF4 plays a unique role in stem cell reprogramming by facilitating the mesenchymal to epithelial transition (MET) [20]. The cellular phenotypic switch from epithelial to mesenchymal cell transition (EMT) is a fundamental process in tumor metastasis that is a prominent feature of ovarian carcinomas. The MET or EMT leads to the alterations of epithelial and mesenchymal marker gene expression that include snail1 & 2, Zeb 1 &2, Twist, vimentin, E-cadherin [18], [21], [22]. EMT is regulated by multiple signaling pathways, which include WNT, TGFβ, and Notch. [23], [24], [25]. Recent studies indicate that miRNAs regulate EMT or MET pathways by targeting epithelial or mesenchymal cell marker genes that include miR-194, miR-203, and miR-200c [22], [26]. KLF4 has been shown to regulate EMT in several different cancer cells. In hepatocellular carcinoma, breast, and prostate cancer cells, KLF4 activates the transcription of the epithelial cell marker gene E-cadherin and represses the mesenchymal cell marker gene snail 2(slug) by binding to their respective promoters. KLF4 in these cancers promotes MET and inhibits tumor cell growth [10], [24], [27]. In the present study, we investigated the role of KLF4 in ovarian cancer cells using a doxycycline (Dox)-dependent KLF4-inducible lentiviral vector (Tet-on) and found that inducible overexpression of KLF4 reduced cell proliferation, migration, and invasion through promoting MET in ovarian cancer cells.

## Materials and Methods

### Cell culture

The ovarian cancer cell lines SKOV3, OVCAR3 and breast cancer cell line MCF7 were obtained from ATCC and cultured in Dulbecco's Modified Eagle Medium (DMEM) supplemented with 10% FBS (Hyclone; Logan, UT), 100 U/ml penicillin, and 100 µg/ml streptomycin (Invitrogen; Carlsbad, CA). HEK293 FT cells were cultured in DMEM media with 10% FBS, 100 U/ml penicillin, 100 µg/ml streptomycin, 1% glutamine, 1% nonessential amino acid, and geneticin with a final concentration of 1 µg/ml.

### Lentiviral vector production

The Dox-inducible KLF4, reverse transactivator (rtTA-M3), and EGFP lentiviral vectors were packaged in HEK293FT cells and produced as described previously [28]. Stable cell lines with overexpression of KLF4- or EGFP- were generated by co-transducing the SKOV3, OVCAR3, and MCF7 cells with the lentiviral vectors KLF4, EGFP with rtTA-M3 and selected with 5 µg/ml puromycin. To induce KLF4 and EGFP expression, Dox was added into normal growth medium as indicated.

### Cell colony formation assay

SKOV3, MCF7 and OVCAR3 cells transduced with KLF4 or EGFP overexpression viruses (200 cells/well each) were plated in triplicate into 6-well plates and cultured for 2 weeks. They were then stained with 0.1% crystal violet, and cell colonies were counted as described previously[29].

### MTT assay

Cells were plated 8000 per well in 96-well plates and cultured for 24 h. Thereafter, 10 µl of MTT reagent were added to each well and incubated for ∼4 h. The reaction was terminated by adding 100 µl detergent reagent; the plates were incubated at 22°C in the dark for 2 h; and then the absorbance was measured at 570 nm wavelength.

### Cell migration assay

Transduced cells (3×10^5^ cells per well) were seeded in triplicate into 6-well plates and cultured for 24 h. The cell surface was scratched with a pipette tip and washed three times with PBS. Fresh growth medium was added for an additional 24 h. The migration rate was calculated using the following formula: (area of the wound area at 0 h - the wound area at 24 h)/the wound area at 0 h. The transwell migration assay was performed using a modified chamber (BD Falcon, San Jose, CA). These chambers were inserted into a 24-well plate. Cells (3×10^4^) in 300 µl serum-free DMEM were added to the upper chamber. The chemoattractant in DMEM was added into the lower chamber of each well and cells were incubated for 24 h. The medium and non-migrated cells in the upper chamber were removed whereas, the migrated cells in the lower side of the membranes were fixed with methanol and stained with crystal violet. Pictures were taken at 10X magnification. Cells in at least three different fields were counted.

### Cell invasion assay

SKOV3 and OVCAR3 cells (5×10^5^) transduced with EGFP and KLF4 overexpression viruses were seeded in serum-free DMEM onto inserts precoated with Matrigel (BD BioCoat, 24-well Tumor Invasion System (BD BioSciences, San Jose, CA). DMEM containing 10% FBS was added to the bottom chamber of the invasion system as the chemoattractant. After 24 h, the transwell inserts were stained using 4 µg/ml of Calcein AM (Life Technologies, Grand Island, NY) at 37°C for 1 h. The fluorescent intensity was measured using the BioTek Synergy (Winooski, VT) plate reader at excitation and emission wavelengths of 485 nm and 528 nm, respectively.

### Soft agar assay

For the bottom agar, 0.6% Noble agar in DMEM containing 10% FBS was added to 6-well plates. Two million cells in DMEM containing 10% FBS and 0.35% Noble agar were added to the bottom agar. Growth medium was then added to the top agar once it had solidified and the cells were fed with fresh growth medium every 5 d, and colonies were counted under a light microscope after 2 weeks.

### Immunofluorescent staining

To detect the expression of EMT-associated marker genes, KLF4-expressing and control SKOV3 cells were fixed for 10 min using 4% PFA, washed three times with 0.1% Tween20 in PBS (PBST), and incubated with blocking buffer (5% normal goat serum, 3% bovine serum albumin, and 0.1% Triton-X 100 in PBS) for 1 h. The primary antibodies to E-cadherin, snail2, and vimentin (1∶200 dilution, Cell Signaling, Danvers, MA), were incubated with fixed cells overnight. After rinsing three times for 5 min with PBST, Alexa 488 or 594 conjugated goat anti-rabbit (1∶200 dilution, Life Technologies) antibodies were added for 1 h at room temperature. Cell nuclei were counterstained with DAPI (Vector Laboratories, Inc.; Burlingame, CA). Images were taken using a Nikon inverted fluorescence microscope.

### Chromatin immunoprecipitation (ChIP)

ChIP was performed using the ChIP-IT Express Enzymatic kit (Active Motif, Carlsbad, CA) according to the manufacturer's instructions. Briefly, KLF4-expressing and control SKOV3 cells were cross-linked with 1% formaldehyde for 10 min at room temperature. Cells were harvested and sonicated to shear chromosomal DNA. Immunoprecipitation was performed by binding 10 µg of rabbit anti-KLF4 antibody or IgG (Santa Cruz Inc., Dallas, Texas) to the lysates and incubated at 4°C with rotation overnight. Chromatin complexes were eluted from magnetic beads by reverse-crosslink. Chromatin DNA was purified using the QIAquick PCR Purification Kit (Qiagen, Valencia, CA) and eluted into 40 µl of water. DNA (4 µl) was used for quantitative PCR reaction to detect the presence of E-cadherin promoter. Primers used to detect the promoter of E-cadherin were 5′-TAG AGG GTC ACC GCG TCT AT-3′ (forward) and 5′-TCA CAG GTG CTT TGC AGT TC-3(reverse) as described previously [16].

### Western blot

Ovarian and breast cancer cells were collected in RIPA buffer (Thermo Scientific; Rockford, IL) containing 1% Halt Proteinase Inhibitor Cocktail (Thermo Scientific; Rockford, IL). An equal amount of protein (40 µg/lane) was loaded onto 10% SDS-PAGE gels and transferred onto nitrocellulose membranes. The membranes were blocked with 5% non-fat milk for 1 h and incubated with primary antibodies against KLF4 (Cell Signaling), GAPDH (Sigma; St. Louis, MO), vimentin, E-cadherin, or snail2 (Cell Signaling).

### Statistical analysis

Significant differences were determined from two or three independent experiments performed in triplicate and presented as means ± S.D. using Student's *t*-test. *p*<0.05 was considered significant.

## Results

### Overexpression of KLF4 in ovarian cancer cells using Tet-on system

To determine the role of KLF4 in ovarian cancer cells, we constructed an inducible lentiviral vector, in which the KLF4 gene was driven by tetracycline- (Tet) or a Dox-responsive promoter (TRE-tight). The reverse transcription activator rtTA-M3 was driven by a constitutive human ubiquitous C promoter cloned into a separate lentiviral vector (Figure S1A). KLF4 expression can be activated by rtTA-M3 in the presence of Dox in a dose-dependent manner. An EGFP-inducible lentiviral vector was constructed and served as control. To examine the induced expression of KLF4 and EGFP in the ovarian cancer cell lines SKOV3 and OVCAR3, Dox was added at a final concentration of 1 µg/ml, and the expression of KLF4 and EGFP at different time points was detected by Western blot. Expression of EGFP and KLF4 increased gradually over a 24 h period (Figure S1B). EGFP expression in SKOV3 and OVCAR3 ovarian cancer cells was visualized using fluorescent microcopy 48 h after the addition of Dox (Fig. S2A, B). Dox treatment caused changes in cell morphology of SKOV3 cells. A rounded epithelial cell-like morphology was observed in KLF4-transduced SKOV3 cells compared to flattened multipolar epithelial cell-like shape of EGFP or KLF4 control cells at 48 and 72 h (Fig. S1C and Fig. S2C). However, the morphology in KLF4-transduced OVCAR3 cells was not altered following Dox treatment compared with control cells (data not shown).

### KLF4 inhibits cell proliferation and colony formation

**Figure 1:**
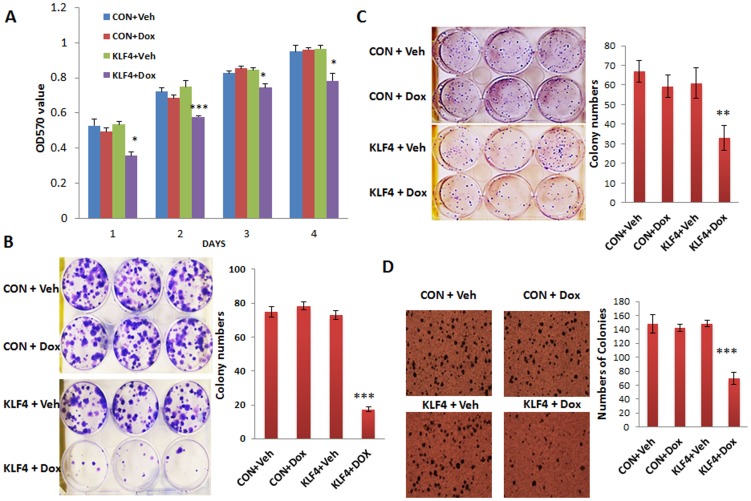
KLF4 inhibits cell proliferation and colony formation. **A**. Cell proliferation of SKOV3 cells transduced with either EGFP or KLF4 was examined by MTT assay at different time points following Dox treatment. Overexpression of KLF4 significantly reduced cell proliferation compared to that in Dox-treated control cells (*p<0.05). **B**. 200 SKOV3 cells transduced with lentiviral KLF4 or EGFP control vectors were seeded into each well of a 6-well plate and cultured for 14 d. Cell colonies were counted following crystal violet staining. The number of colonies in KLF4-overexpressing cells was significantly reduced compared to that in Dox-treated control cells (***p<0.001). **C**. The number of colonies in KLF4-overexpressing OVCAR3 cells was significantly reduced compared to that in Dox-treated control cells (**p<0.01). **D**. Soft agar colony formation assay was performed in triplicate using SKOV3 cells. Colonies were photographed and counted after 3 weeks. The number of colonies in KLF4-overexpressing cells was significantly reduced compared to that in Dox-treated control cells (***p<0.001).

To investigate the role of KLF4 in ovarian cancer cell proliferation, the MTT assay was performed in KLF4-transduced SKOV3 and control cells. Following Dox treatment, proliferation in KLF4-overexpressing cells was significantly inhibited compared to EGFP or KLF4 (non-Dox) control cells (Figure 1A). We also performed colony formation assays on SKOV3 and OVCAR3 cells transduced with KLF4 and EGFP control vectors. The number of colonies in KLF4-overexpressing cells was significantly reduced compared to EGFP and KLF4 control cells (Figure 1B, C). In addition, soft agar colony formation assays were also performed to determine whether KLF4 affects anchorage-independent cell growth. Similarly, our results indicated that KLF4 inhibits cell proliferation in the semisolid culture media compared to control cells (Figure 1D).

### KLF4 reduces cell migration and invasion

**Figure 2:**
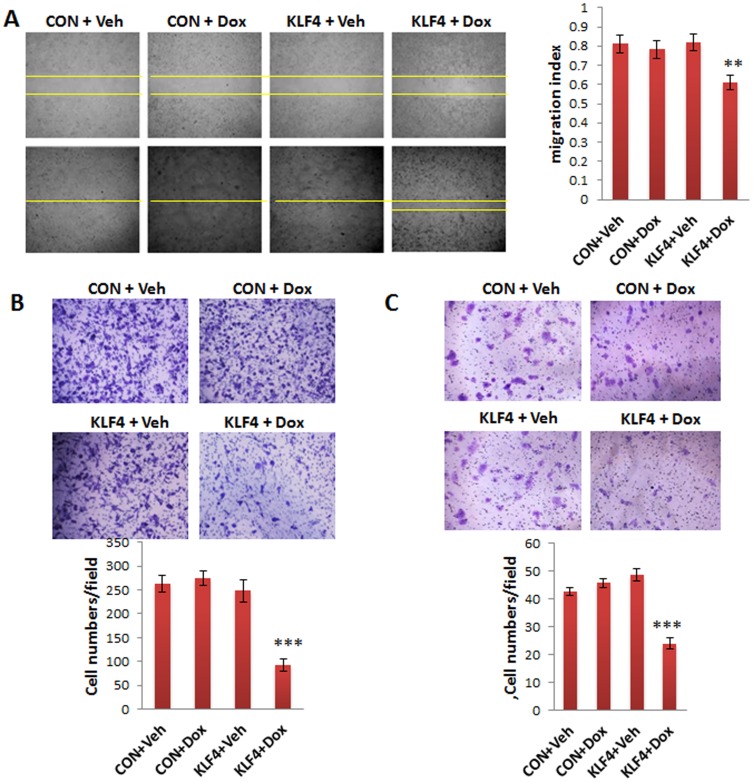
KLF4 reduces cell migration in ovarian cancer cells. **A**. Wound-healing assay was performed to examine the migration rate of SKOV3 cells transduced with KLF4 and EGFP lentiviral vectors. Photographs were taken at 0 and 24 h following the initial scratch. Migration rates were quantified by measuring three different wound areas. Three separate experiments were performed. Migration rate was significantly reduced in KLF4-overexpressing cells compared to that in Dox-treated controls (**p<0.01). **B, C**. Transwell migration assay was performed in SKOV3 and OVCAR3 cells. Overexpression of KLF4 significantly reduced cell migration in SKOV3 (**B**) and OVCAR3 (**C**) cells compared with that in Dox-treated controls (***p<0.001).

**Figure 3:**
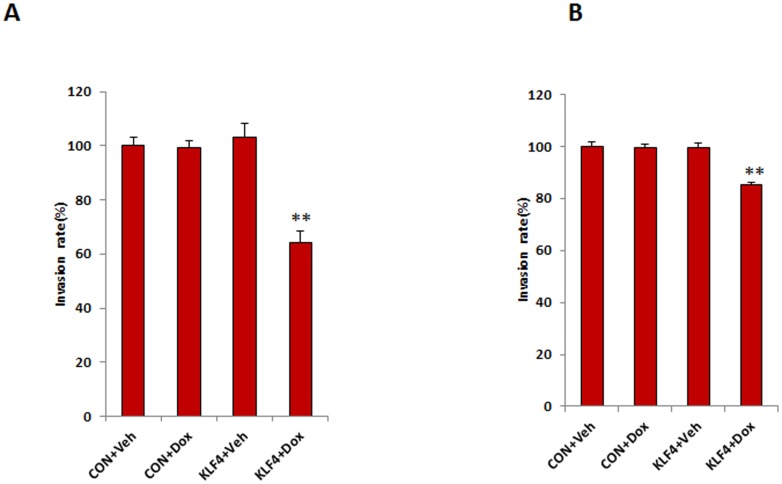
KLF4 reduces ovarian cancer cell invasion. **A, B**. Cell invasion assay was performed using Matrigel-coated transwell plates for SKOV3 (**A**) and OVCAR3 cells (**B**). The invasion rate was significantly reduced in KLF4-overexpressing cells compared to that in Dox-treated control cells from both SKOV3 and OVCAR3 cells (**p<0.01). Data were collected from three separate experiments and analyzed using Student *t*-tests.

One crucial property of invasive cancer cells is their increased mobility. To investigate whether KLF4 affects cell migration in the ovarian cancer cell line SKOV3, wound-healing assay was performed using KLF4-transduced SKOV3 and control cells. As shown in Figure 2A, the cell migration rate was significantly reduced in KLF4-overexpressing cells compared to rates in EGFP- and KLF4-transduced control cells. Cell chemotaxis was examined using the transwell migration assay. As shown in Figure 2B and C, migrated cells were significantly reduced in KLF4-overexpressing SKOV3 and OVCAR3 cells compared to cells EGFP and KLF4 controls. To examine whether KLF4 affects cell invasion, a cell invasion assay was performed on KLF4-transduced SKOV3 and OVCAR3 cells using Matrigel-coated transwell plates. Overexpression of KLF4 significantly reduced cell invasion compared to that in controls in both cell lines (Figure 3A, B).

### KLF4 promotes MET in ovarian cancer cells

**Figure 4:**
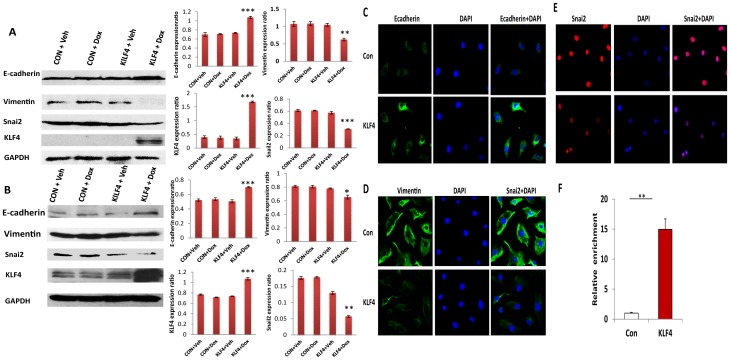
KLF4 promotes mesenchymal-epithelial cell transition. **A. B**. Western blots were performed in KLF4- and EGFP-transduced SKOV3 (**A**) and OVCAR3 cells (**B**) with or without Dox induction. E-cadherin expression was significantly upregulated (***p<0.001), whereas vimentin (**p<0.01) and snail2 (***p<0.001) were downregulated in KLF4- overexpressing SKOV3 cells compared to control cells (**A**). E-cadherin (**p<0.01) was upregulated, whereas vimentin (*p<0.05) and snail2 (***p<0.001) were downregulated in KLF4-overexpressing OVCAR3 cells compared to Dox-treated control cells (**B**). E-cadherin (**C**) and vimentin (**D**) were immunostained in cellular membranes in KLF4-expressing and control SKOV3 cells. **E**. Snail2 was stained in cell nuclei in KLF4-expressing and control SKOV3 cells. **F**. KLF4 binding to the promoter of E-cadherin in SKOV3 cells was examined by chromatin immunoprecipitation using KLF4 antibody and detected by real-time PCR using E-cadherin-specific primers. The ChIP-enriched DNA levels were normalized to input DNA, followed by subtraction of non-specific binding determined by control IgG (***p<0.001).

To investigate whether KLF4 regulates EMT in ovarian cancer cells, the epithelial cell marker gene E-cadherin and mesenchymal marker genes snail2 and vimentin were examined in KLF4-transduced SKOV3 and OVCAR3 cells using Western blot. The epithelial marker E-cadherin was significantly upregulated, whereas the mesenchymal markers vimentin and snail2 were significantly reduced in KLF4-overexpressing cells compared to EGFP and KLF4 controls (Figure 4A, B). We also performed immunostaining on KLF4-overexpressing and control cells to examine MET markers. Both E-cadherin and vimentin staining were prominent in cellular membranes, whereas snail2 stained the cell nuclei. Similarly to the Western blots, immunostaining showed that E-cadherin expression was upregulated, whereas vimentin and snail2 were downregulated (Figure 4C, D, and E). We also examined the expression of twist1 using realtime RT-PCR in KLF4 transduced SKOV3 cells and found that twist1 was significantly downregulated in KLF4 expressing SKOV3 cells compared with control (Fig. S4). These results support the hypothesis that KLF4 promotes MET in ovarian cancer cells. To further examine whether E-cadherin upregulation was caused by transcriptional activation, we performed chromatin immunoprecipitation in KLF4-expressing and control SKOV3 cells, and the promoter region of E-cadherin was amplified from enriched chromatic DNA by real-time PCR as described previously [16]. KLF4 expression in SKOV3 cells led to an approximately 15-fold enrichment of E-cadherin compared to controls (Figure 4F), indicating that KLF4 binds to the promoter of E-cadherin and activates E-cadherin expression in ovarian cancer cells.

### KLF4 inhibits TGFβ-induced EMT in ovarian cancer cells

**Figure 5:**
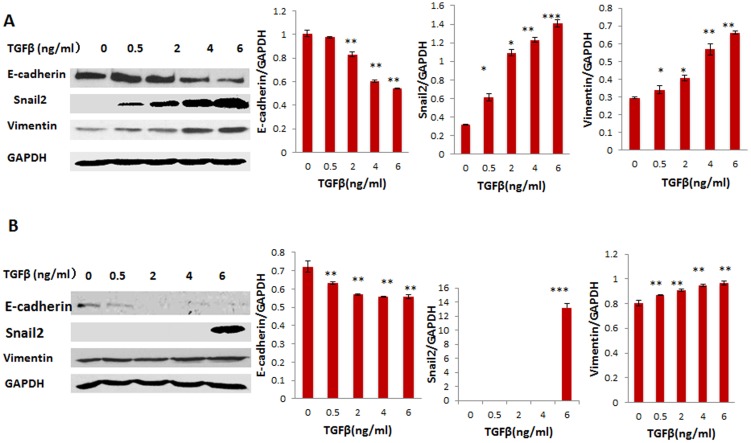
TGFβ promotes EMT in ovarian cancer cells. Human ovarian cancer cell lines SKOV3 (**A**) and OVCAR3 (**B**) were treated with different doses of TGFβ for 48 h. EMT-associated marker genes, including E-cadherin, snail2, and vimentin were examined using Western blot. Significances were determined by comparing TGFβ treated to non-treatment (*p<0.05, **p<0.01, ***p<0.001).

**Figure 6:**
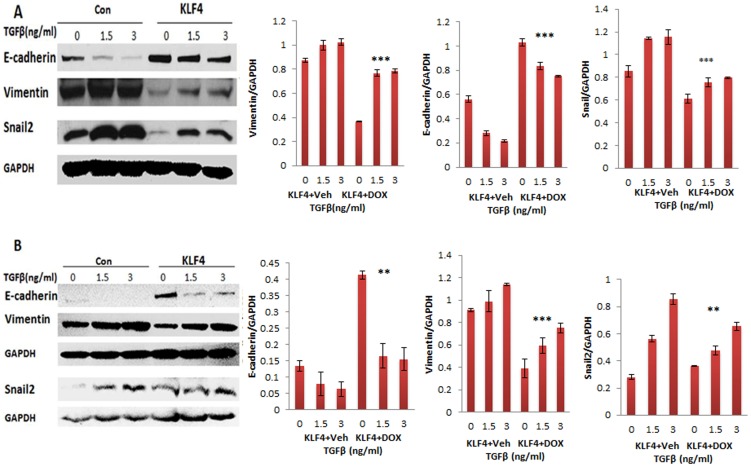
KLF4 inhibits TGFβ-induced EMT in ovarian cancer cells. Ovarian cancer cell lines SKOV3 (**A**) and OVCAR3 (**B**) transduced with lentiviral KLF4 overexpression and control vector were treated with TGFβ for 48 h, and the protein expressions of EMT-associated marker genes E-cadherin, snail2 and vimentin were examined using Western blot. Significant differences were compared between KLF4 expressing and control group (**p<0.05, ***p<0.001).

To examine the mechanism whether KLF4 regulates TGFβ induced EMT in ovarian cancer cells, SKOV3 and OVCAR3 cells were treated with different doses of TGFβ. Expression of the EMT marker proteins E-cadherin, vimentin, and snail2 was examined using Western blot. Our data indicate that TGFβ promoted EMT in ovarian cancer cells by downregulating E-cadherin and upregulating snail2 and vimentin (Figure 5A, B). Furthermore, when we treated KLF4-expressing and control SKOV3 and OVCAR3 cells with increasing doses of TGFβ, KLF4 expression significantly inhibited TGFβ-induced EMT in both cell lines (Figure 6A, B).

## Discussion

In this study, we investigated the role of KLF4 in ovarian cancer cells using lentiviral vector mediated inducible expression. Previous studies showed that KLF4 was downregulated in ovarian cancers compared to controls and that KLF4 did not affect cell proliferation but increased the Bcl-2/Bax ratio and inhibited apoptosis [19]. In contrast, we found that KLF4 inhibits the proliferation of SKOV3 cells in colony formation and MTT assays (Fig. 1A, B, and C). The discrepancy may be caused by the low transfection efficiency in that study compared to our highly efficient inducible lentiviral transduction. We also found that KLF4 inhibits cell proliferation in OVCAR3 cells (Fig. 1C). Our data demonstrate that KLF4 inhibits cell proliferation, migration, and invasion, hence it functions as a tumor suppressor in both SKOV3 and OVCAR3 cells.

KLF4 can have a dual effect either as an oncogene or a tumor suppressor in breast cancer cells [14], [16], [18], [30]. To compare the actions of KLF4 in MCF7 breast cancer with that in ovarian cancer cells, we performed similar experiments using the same lentiviral Tet-on inducible vector-transduced in MCF7 cells. We found that KLF4 inhibits MCF7 cell proliferation (Fig. S3A). Our finding is similar to the results shown in MCF10A cells by Yori et al. [16]. Yu et al., however, showed that KLF4 functions as an oncogene in MCF7 cells [14], which is opposite to our finding.

The morphology in KLF4 expressing SKOV3 cells in the presence of Dox was clearly altered from control cells without Dox at the different time points (Fig S2C); however, we did not observe obvious morphological alteration in OVCAR3 cells following induction of KLF4 expression. One of reasons causing this phenomenon is that KLF4 also induced cell apoptosis in ovarian cancer cells based on our unpublished data. The morphological differences may be caused by the different responses to KLF4 induced apoptosis in both cell lines. In both SKOV3 and OVCAR3 cells the epithelial cell marker gene E-cadherin was upregulated, and the mesenchymal marker genes vimentin and snail2 were downregulated following induction of KLF4 overexpression (Fig. 4A, B). Altogether, these data suggest that KLF4 primarily promoted MET in ovarian cancer cells. The expression of EMT associated marker genes in ovarian cancer cells are similar to what we observed in MCF7 breast cancer cells, although the endogenous expression of vimentin in MCF7 was not detectable in KLF4-overexpressing or control cells. This may be caused by low endogenous expression level of vimentin in MCF7 cells. Our data support the hypothesis that KLF4 is a key regulator promoting MET and inhibiting EMT in ovarian and breast cancer cells.

Previous studies showed that KLF4 binds to the promoter regions of E-cadherin and snail2, thus can activate the expression of E-cadherin or repress the expression of snail2 in fibroblasts and several cancer cell lines [18], [24], [27], [31]. The expression of epithelial cell marker E-cadherin is required for stem cell reprogramming. KLF4 facilitates the MET process by activating E-cadherin expression [31]. In ovarian cancer cells, we observed a KLF4-dependent upregulation of E-cadherin and a downregulation of vimentin and snail2. Our data indicate that KLF4 transcriptionally binds to the promoters of E-cadherin, thus leading to the activation of E-cadherin expression in ovarian cancer cells.

The EMT or MET is tightly regulated by multiple signaling pathways. Several studies have shown that multiple signaling pathways, including WNT, Notch, NFkB, and TGFβ, are involved in EMT or MET transition in cancers [32], [33], [34], [35]. Previous studies also showed that TGFβ promotes EMT in ovarian cancer cells [36], [37]. However, there are no related studies on describing the mechanism how KLF4 is involved in EMT and interacts with those pathways in ovarian cancer cells. Our current studies indicate that KLF4 inhibits TGFβ-induced EMT in both SKOV3 and OVCAR3 cells (Fig. 6A, B), suggesting that KLF4 attenuates the TGFβ induced EMT in ovarian cancers. Therefore, synergistically overexpression of KLF4 and inhibition of TGFβ pathway will provide a novel approach in the developing new therapeutic drugs for the treatment of ovarian cancers.

In summary, this is the first report showing that KLF4 functions as a tumor suppressor by inhibiting cell proliferation, migration and invasion in ovarian cancer cells through attenuating TGFβ-induced EMT.

## Supporting Information

Figure S1 **Induction of KLF4 expression in ovarian cancer cells using lentiviral Tet-on vector.** **A**. Lentiviral Tet-on vector system. Reverse transactivator (rtTA-M3) was driven by human ubiquitin C (UBC promoter), and EGFP or KLF4 was driven by the Dox inducible promoter TRE-tight. To induce the expression of EGFP or KLF4, Dox is required to activate the Tet promoter following rtTA binding to the Tet-responsive element in the promoter region. **B**. EGFP and KLF4 expressions were induced by Dox in SKOV3 ovarian cancer cells and detected by Western blot. **C**. SKOV3 cells overexpressing KLF4 display rounded epithelial cell-like morphology.
(PDF)

Figure S2 **Dox-induced EGFP expression in SKOV3 and OVCAR3 cells.** EGFP expressions in SKOV3 (**A**) and OVCAR3 cells (**B**) transduced with EGFP lentiviral vector were visualized under fluorescent microscopy with or without Dox induction. Cell morphologies were examined under light microscopy. **C**. Cell morphologies were imaged at different time points under light microscopy.
(PDF)

Figure S3 **KLF4 promotes MET in breast cancer MCF7 cells.** **A**. Colony formation was performed in MCF7 cells transduced with EGFP and KLF4 overexpression lentiviral vectors. The number of colonies in KLF4-overexpressing cells was significantly reduced compared to that in Dox-treated controls (***p<0.001). **B**. Western blot analysis of KLF4 (**p<0.01), E-cadherin (**p<0.01), and snail2 (*p<0.05) in MCF7 cells overexpressing KLF4 and EGFP with or without Dox treatment.
(PDF)

Figure S4 **KLF4 downregulates twist1 expression in ovarian cancer SKOV3 cells.** Twist1 expression in KLF4 expressing SKOV3 and control cells was detected by real time RT-PCR following KLF4 induction for 24 h using 1 ug/ml of doxycycline (*p<0.05).
(PDF)

Text S1 (DOCX)
