# Supplementary figures and images for: Doxycycline Inducible Kruppel-Like Factor 4 Lentiviral Vector Mediates Mesenchymal to Epithelial Transition in Ovarian Cancer Cells
Source: PLoS One. 2014 Aug 19;9(8):e105331. doi: 10.1371/journal.pone.0105331 (PMC4138168; doi:10.1371/journal.pone.0105331)

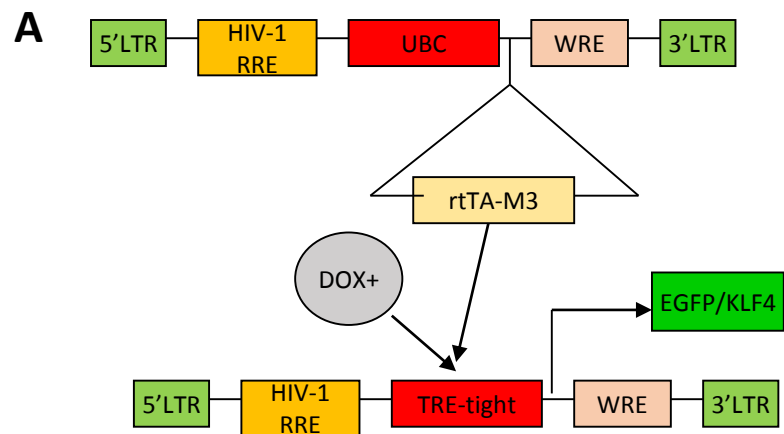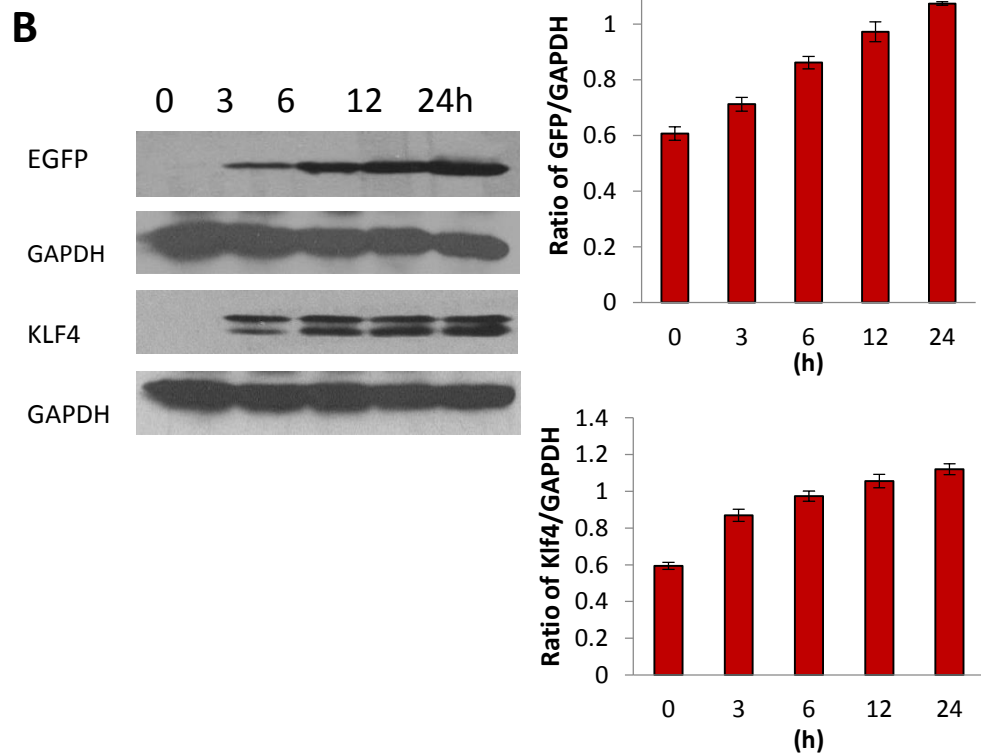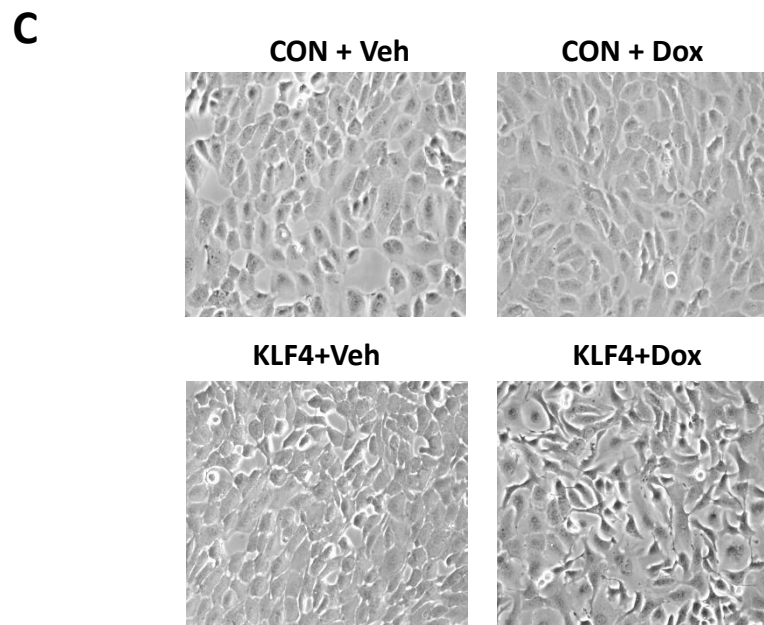

Supplement: Figure S1 — Induction of KLF4 expression in ovarian cancer cells using lentiviral Tet-on vector. A. Lentiviral Tet-on vector system. Reverse transactivator (rtTA-M3) was driven by human ubiquitin C (UBC promoter), and EGFP or KLF4 was driven by the Dox inducible promoter TRE-tight. To induce the expression of EGFP or KLF4, Dox is required to activate the Tet promoter following rtTA binding to the Tet-responsive element in the promoter region. B. EGFP and KLF4 expressions were induced by Dox in SKOV3 ovarian cancer cells and detected by Western blot. C. SKOV3 cells overexpressing KLF4 display rounded epithelial cell-like morphology. (PDF) [file pone.0105331.s001.pdf]

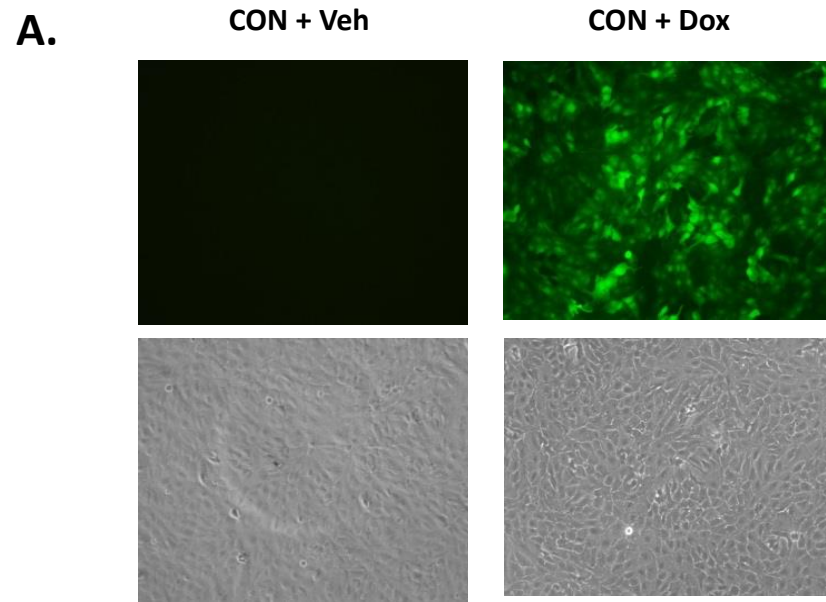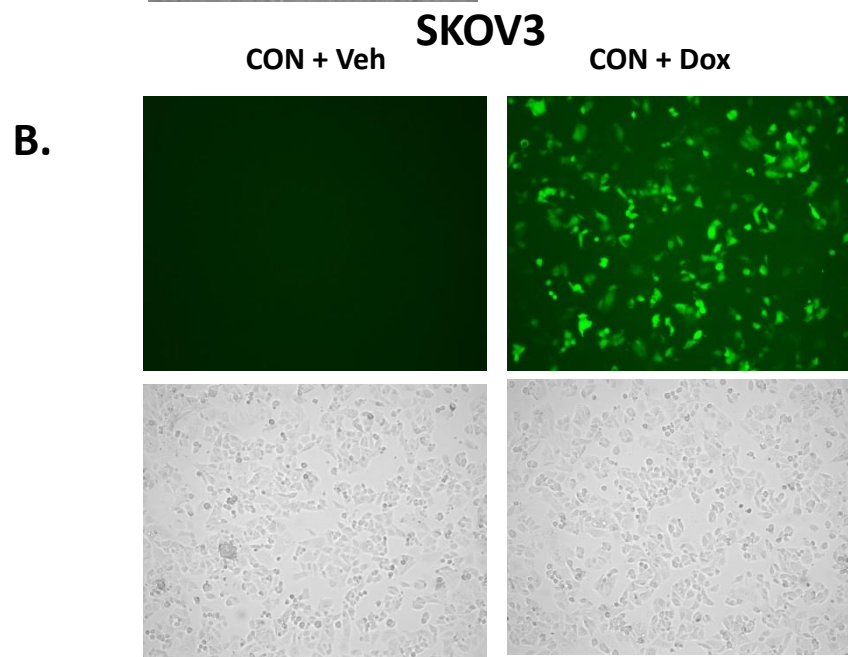

**OVCAR3**

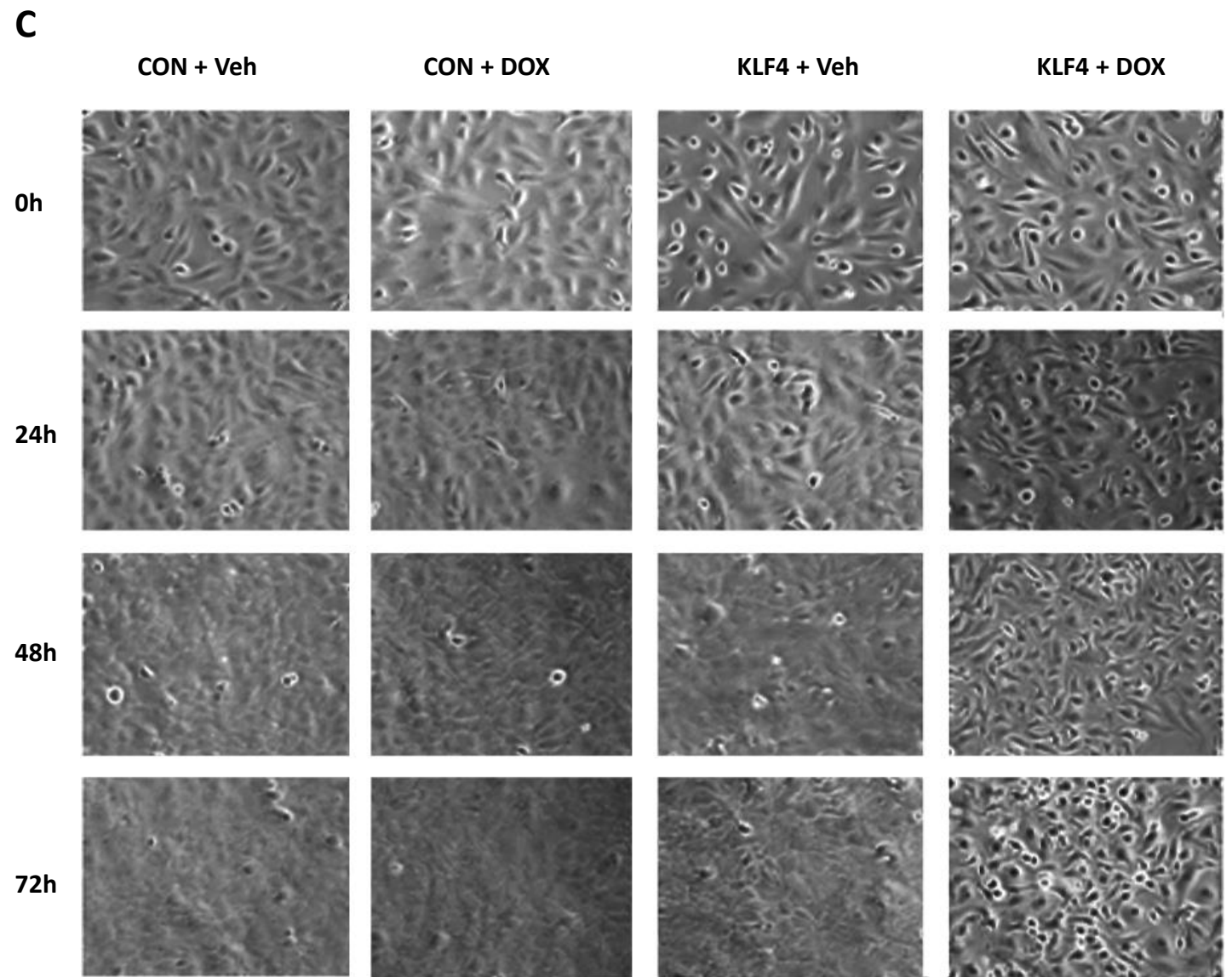

Supplement: Figure S2 — Dox-induced EGFP expression in SKOV3 and OVCAR3 cells. EGFP expressions in SKOV3 (A) and OVCAR3 cells (B) transduced with EGFP lentiviral vector were visualized under fluorescent microscopy with or without Dox induction. Cell morphologies were examined under light microscopy. C. Cell morphologies were imaged at different time points under light microscopy. (PDF) [file pone.0105331.s002.pdf]

**A**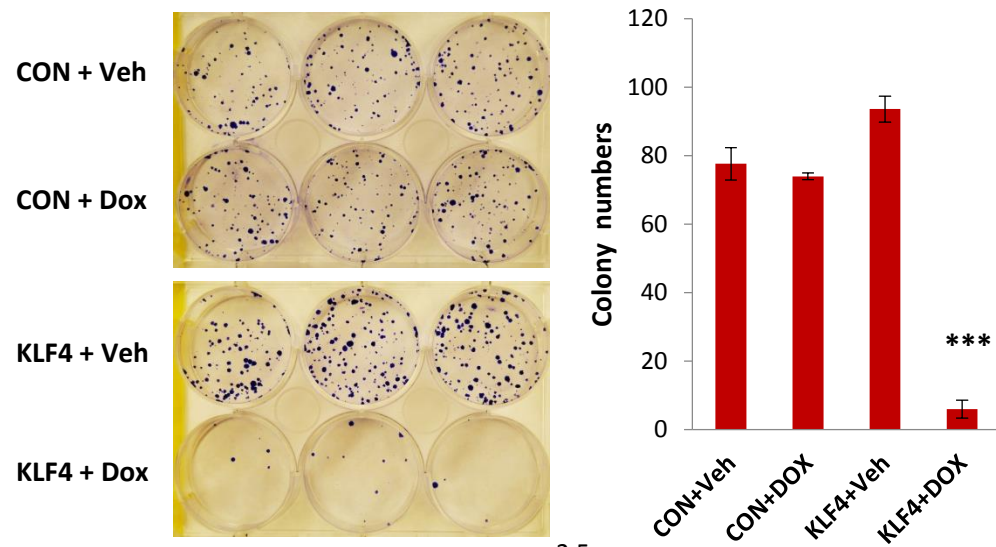**B**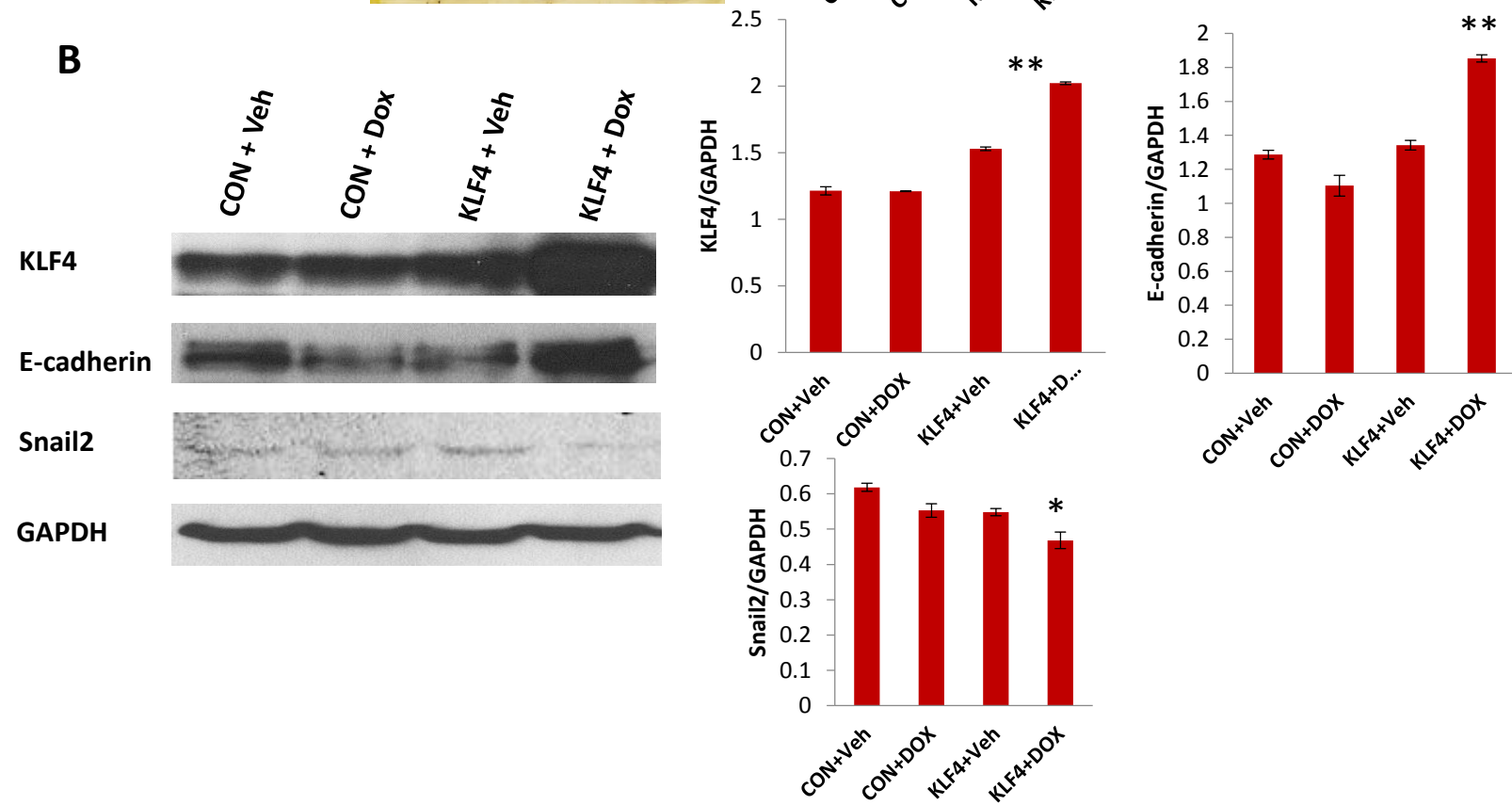

Supplement: Figure S3 — KLF4 promotes MET in breast cancer MCF7 cells. A. Colony formation was performed in MCF7 cells transduced with EGFP and KLF4 overexpression lentiviral vectors. The number of colonies in KLF4-overexpressing cells was significantly reduced compared to that in Dox-treated controls (***p<0.001). B. Western blot analysis of KLF4 (**p<0.01), E-cadherin (**p<0.01), and snail2 (*p<0.05) in MCF7 cells overexpressing KLF4 and EGFP with or without Dox treatment. (PDF) [file pone.0105331.s003.pdf]

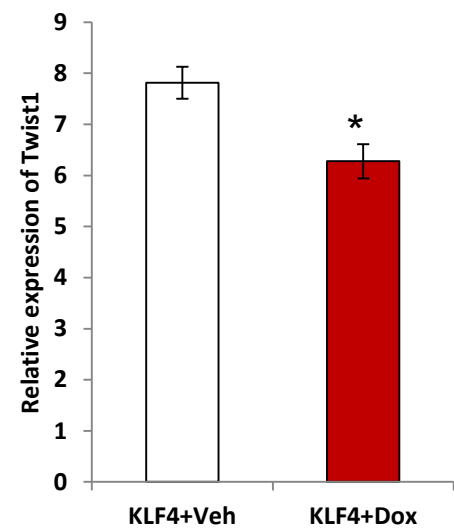

Supplement: Figure S4 — KLF4 downregulates twist1 expression in ovarian cancer SKOV3 cells. Twist1 expression in KLF4 expressing SKOV3 and control cells was detected by real time RT-PCR following KLF4 induction for 24 h using 1 ug/ml of doxycycline (*p<0.05). (PDF) [file pone.0105331.s004.pdf]
