# Supplementary material for: Doxycycline Inducible Kruppel-Like Factor 4 Lentiviral Vector Mediates Mesenchymal to Epithelial Transition in Ovarian Cancer Cells
Source: PLoS One. 2014 Aug 19;9(8):e105331. doi: 10.1371/journal.pone.0105331 (PMC4138168; doi:10.1371/journal.pone.0105331)
Supplement: Text S1 — (DOCX) [file pone.0105331.s005.docx]

**Supplemental Methods:**

*Real time RT-PCR*- RNA was isolated from KLF4 expressing SKOV3 and control ovarian cancer cells using the RNeasy Mini kit (Qiagen, Valencia, CA) according to manufacturer’s instruction. One microgram of total RNA was converted to cDNA with RevertAid First Strand cDNA Synthesis Kit (Thermo Scientific, Rockford, IL ) and Quantitative PCR were performed using 0.6 μl of the cDNA with 300 nM of each primer in a final volume of 25 μl containing 2 x Maxima SYBR Green/ROX qPCR master mix (Thermo Scientific, Rockford, IL). The cycling conditions were 10 min at 95 °C, followed by 40 cycles of 94°C for 15 s and 60°C for 60 s with StepOnePlus real-time PCR system (Applied Biosystems, Grand Island, NY). The relative Twist1 expression in KLF4 expressing SKOV3 (Dox) compared to control cells (non-Dox) was determined using the comparative Ct method (ΔΔCt) by normalizing to endogenous GAPDH expression. Primers used for amplifying twist 1 and GAPDH were 5’-GAGTCCGCAGTCTTACGAGG-3’ (Twist1 forward) and 5’-CTGCCCGTCTGGGAATCACT-3’(twist1 reverse); CTGCACCACCAACTGCTTAG(GAPDH forward), and GGGCCATCCACAGTCTTCT(GAPDH reverse).

**Supplemental Results:**

**KLF4 functions as a tumor suppressor in breast cancer MCF7 cells by promoting MET**

KLF4 has been reported to play a role as both an oncogene and a tumor suppressor in breast cancer cells [[14](#_ENREF_14),[16](#_ENREF_16)]. Because of contradictory reports on the role of KLF4 in breast cancer cells, we decided to examine whether KLF4 plays a similar role in breast cancer cells compared to ovarian cancer cells. We performed similar studies by upregulating KLF4 expression in MCF7 breast cancer cells and control cells using Dox treatment. Overexpression of KLF4 led to a significant reduction in the number of colonies in these cells compared to EGFP and KLF4 control cells (Figure. S3A). Expression of the epithelial marker E-cadherin was also significantly upregulated, whereas the mesenchymal marker snail2 was significantly reduced in KLF4-overexpressing cells compared to EGFP and KLF4 control cells (Figure S3B). However, expression of the mesenchymal marker vimentin was not detectable in KLF4-overexpressing cells and control cells. Our data are consistent with the hypothesis that KLF4 promotes MET or inhibits EMT and functions as a tumor suppressor in breast cancer MCF7 cells.
